# Egocentric Social Network Structure, Health, and Pro-Social Behaviors in a National Panel Study of Americans

**DOI:** 10.1371/journal.pone.0036250

**Published:** 2012-05-15

**Authors:** A. James O’Malley, Samuel Arbesman, Darby Miller Steiger, James H. Fowler, Nicholas A. Christakis

**Affiliations:** 1 Department of Health Care Policy, Harvard Medical School, Boston, Massachusetts, United States of America; 2 Ewing Marion Kauffman Foundation, Kansas City, Missouri, United States of America; 3 Gallup Government, Cleveland, Ohio, United States of America; 4 Departments of Medical Genetics and Political Science, University of California San Diego, La Jolla, California, United States of America; 5 Department of Sociology, Harvard University, Cambridge, Massachusetts, United States of America; University of Maribor, Slovenia

## Abstract

Using a population-based, panel survey, we study how egocentric social networks change over time, and the relationship between egocentric network properties and health and pro-social behaviors. We find that the number of prosocial activities is strongly positively associated with having more friends, or an increase in degree, with approximately 0.04 more prosocial behaviors expected for every friend added. Moreover, having more friends is associated with an improvement in health, while being healthy and prosocial is associated with closer relationships. Specifically, a unit increase in health is associated with an expected 0.45 percentage-point increase in average closeness, while adding a prosocial activity is associated with a 0.46 percentage-point increase in the closeness of one’s relationships. Furthermore, a tradeoff between degree and closeness of social contacts was observed. As the number of close social contacts increases by one, the estimated average closeness of each individual contact decreases by approximately three percentage-points. The increased awareness of the importance of spillover effects in health and health care makes the ascertainment of egocentric social networks a valuable complement to investigations of the relationship between socioeconomic factors and health.

## Introduction

Although egocentric network studies – wherein a subject is asked to identify his or her social contacts and their relationships – have a long history in sociology, their use in health surveys is rare. But increased attention to the role of social networks in medicine suggests that a basic understanding of the structure of American social networks and how they change may be important [1]. We collected egocentric social network data from a nationally representative sample of Americans in order to study the relationship between individuals social networks and their health and related social behaviors. The norm in national population surveys is to sample new individuals each year [2], [3], [4]. While this can allow statistical understanding of population-level, egocentric social networks structure at a fixed time, it does not allow us to study the *change* in individual networks over time. Therefore, here, we also sought to characterize how individuals egocentric social networks change over time, the extent to which close social contacts are gained or lost, and the extent to which an ego’s social contacts (their “alters”) come to know each other over time.

Obtaining social network information that is population representative is challenging. To sample individuals who are actually connected to each other, non-random methods are needed, or else a set of null relationships in which no two individuals know each other is likely [5]. On the other hand, the necessity of sampling pairs of individuals who are connected is at odds with the requirements of population representativeness that are fundamental to most surveys, for which random or probability-weighted random sampling is desirable. Therefore, we implemented a compromise between a population-representative survey and a full sociocentric network ascertainment, in order to obtain population representative estimates of quantities related to networks and various behaviors.

That is, we conducted a national, egocentric study. We fielded a network survey instrument in a nationally representative sample in order to study the relationship between individuals social networks and their health and behaviors. The instrument extracts information from respondents (egos) on their relationships to the peers they spend the most time with or discuss important issues with (alters) and also on the relationships between all pairs of named peers. Moreover, we collected this information repeatedly across time. Thus a respondent’s social network is (partially) revealed in a traditional random sample without having to ascertain the full population network.

Using such a survey instrument, we can examine the change in the number of close social contacts (“degree”), tie strength (“closeness”), and the number of interconnections between contacts (“transitivity”), how they are related to each other, and how they relate to measures of health and prosocial behavior. We focused primarily on prosocial behavior, broadly defined as altruism and generosity, or any activity that promotes the general welfare of society, e.g., participation in community enrichment, contributions to charity, and volunteering [6].

## Methods

### Survey Instrument

We developed a social network survey instrument for deployment with Gallup’s ongoing, longitudinal, probability-based panel of American households. We constructed the survey by modifying the social network instrument used previously by the GSS [7], [8], [9] and by the National Health and Social Life Survey [10], [11]. The survey consists of items seeking information on ego characteristics, alter characteristics, ego-alter relationships, and the relationship between each pair of alters. Ego health and behavioral traits are also measured. Surveys were completed online by the egos.

The network component of the survey asked egos to name (up to) four adults with whom they spent the most free time in the past 12 months and (up to) four adults with whom they most often discussed important issues. Alters could be family members, friends, work/school colleagues, and so forth. Thus, each ego named up to eight distinct alters yielding a maximum of 28 alter-alter relationships for which they also provided information.

Individual characteristics included gender, age, race, ethnicity, education, employment status, income, marital status, religious preference, political affiliation, health, health behaviors, and prosocial behavior. Ego-alter variables included the type of relationship and frequency of contact, and alter-alter variables consisted of type and strength of the relationship. The key alter-alter variable is the ego’s assessment of the strength of their relationship.

The health and behavioral traits for the ego consisted of a series of items that, for analytic purposes, were combined into scales. Prosocial behavior measured whether the ego donated blood, donated money, donated clothing, financially supported a political candidate, or volunteered to help prepare for a major public emergency. Health behaviors included smoking status, BMI (weight/height^2^), whether they wanted to gain or lose weight, whether they took active steps to improve their health (e.g., adhered to a diet, quit smoking, cut back alcohol). The questions on health included physical health, mental health, and missed work due to sickness.

The network measures included the number of alters with whom the ego named as having a close relationship (degree), the average strength of these relationships (closeness), and the connectedness of the named alters to each other according to the ego (transitivity).

### Study Design

The Gallup Panel is a nationally representative, multi-mode panel recruited through random digit dialing methods. Only the web-based portion of the Panel was eligible for participation in this study. Data was collected on randomly chosen and nationally representative Americans in June 2009, December 2009, and July 2010.

A total of 6,000 randomly selected web-based members of the Gallup panel were sent an email invitation that asked them to respond to a survey about “the various people that you spend your free time with and have important conversations with.” Invitations for waves 2 and 3 explained that this was a continuation of the earlier research in which they had participated. A reminder email was sent for each wave in order to boost completion rates. The first wave collected data from a sample of 3,232 respondents (out of 6,000– a 53.9% completion rate). Of those 3,232 respondents, 2,305 responded to wave 2 in December 2009 (71.3%) and 2,114 responded to wave 3 in July 2010 (65.4% of wave 1).

The analysis sample consists of all individuals that responded at wave 1 and, therefore, includes some who have missing data in waves 2 and 3.

### Network Measures

**Figure 1:**
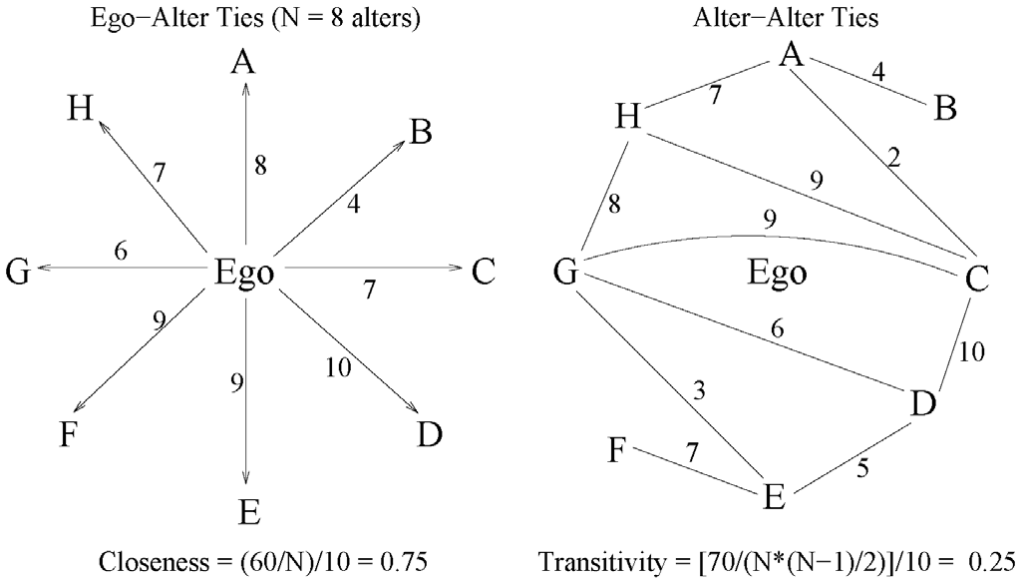
Egocentric network involving an ego with N = 8 alters labeled A, B, …, H. For clarity, the left panel shows only the ego-alter ties while the right panel shows only alter-alter ties. Closeness is computed as the average strength of the ego-alter ties (left panel) and dividing by 10 to make the range 0 to 1. Analogously, transitivity is computed as the average strength of the alter-alter ties (right-panel, including the 0 strength null ties that are not depicted) and dividing by 10.

The *degree* of each ego is the number of alters they name, a quantity ranging from 0 to the maximum 8. The strength of each ego-to-alter and alter-to-alter relationship is rated from 0 =  no-relationship to 10 =  very strong relationship; 0 strength only occurs in alter-to-alter relationships. We scaled degree and strength to obtain quantities that range from 0 to 1; the *closeness* of an ego’s relationships is, therefore, the average strengths of their reported ties (see Figure 1). We hypothesized that closeness would have greater discriminatory power than degree.

**Figure 2:**
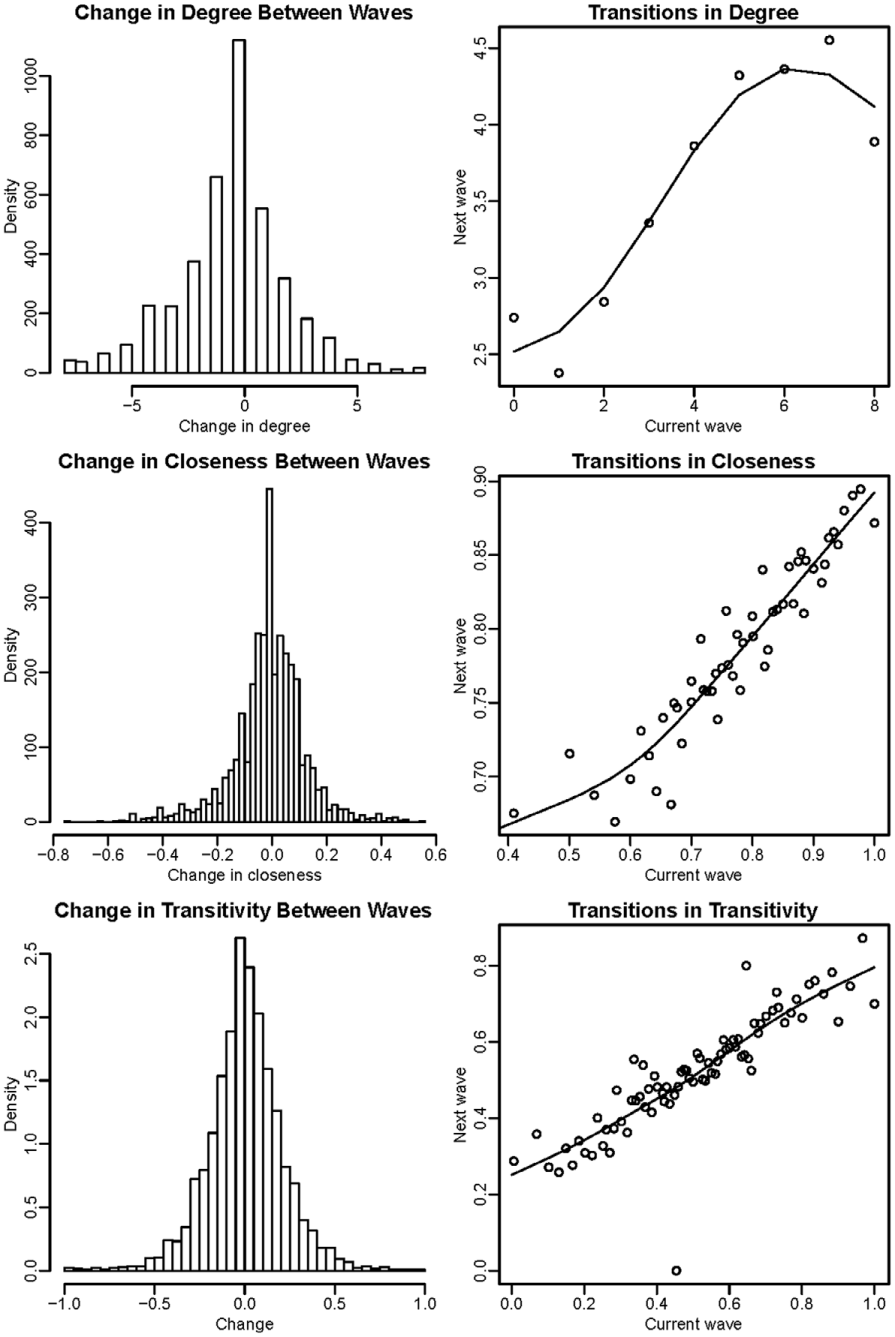
Changes and transitions in network measures between consecutive waves.

**Figure 3:**
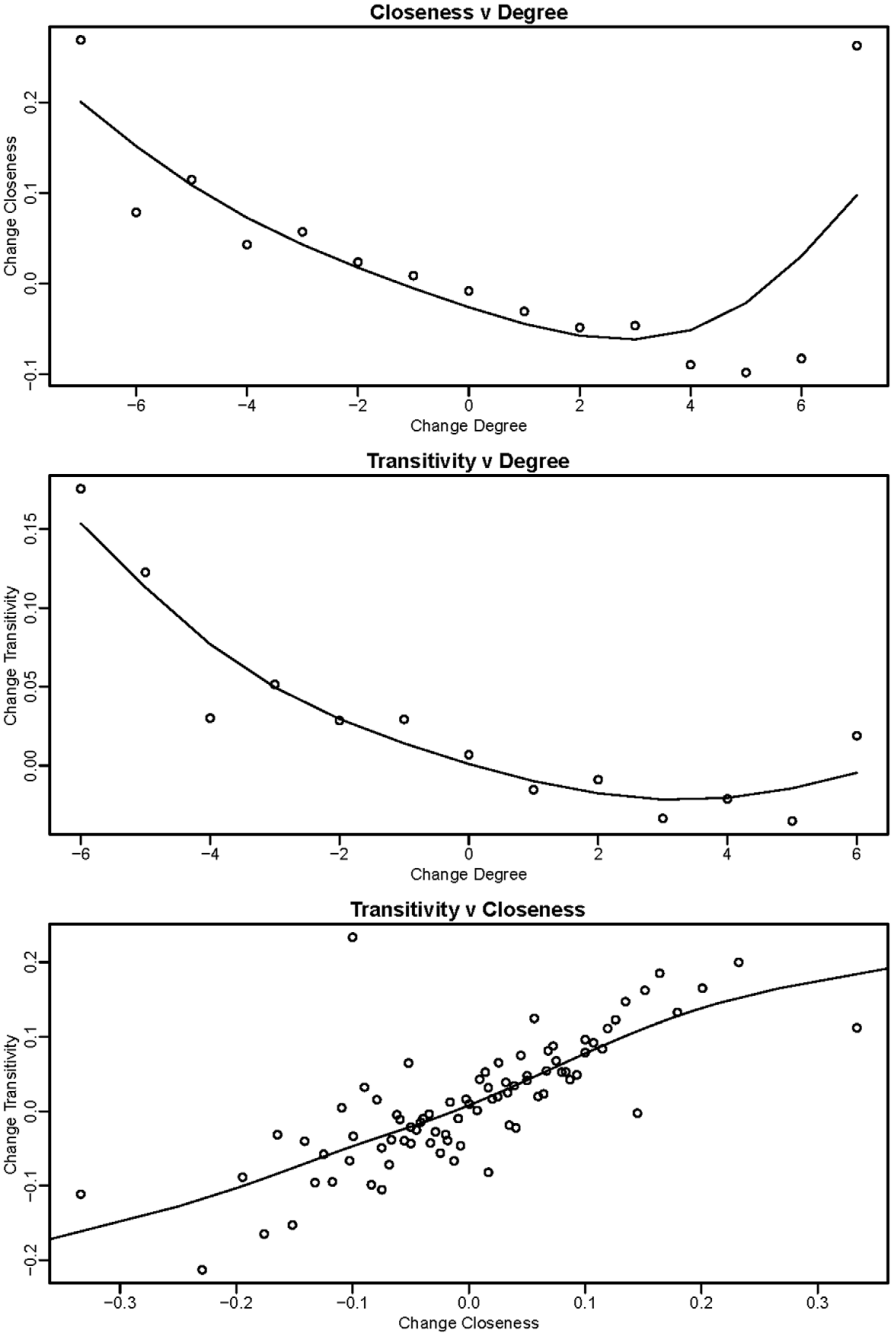
Relationships between changes in network measures.

In egocentric networks, an ego’s transitivity is the average value of the relationship between all pairs of alters, herein assumed to be mutual (i.e., the relationship from *j* to *k* is the same as that from *k* to *j*). Denote the degree of ego *i* by 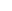 and the strength of the relationship between alter *j* and alter *k* by 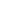 When relationships are binary-valued (e.g., corresponding to the presence of any relationship at all), transitivity is given by
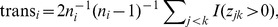
(1)and is interpreted as the proportion of pairs of alters for which some form of relationship exists. If relationships are quantified in terms of their strengths, transitivity is given by



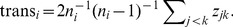
(2)Because (2) is our preferred measure, from here on by “transitivity” we mean the weighted form in (2). Egocentrically, transitivity is a local measure specific to an ego, not a network property.

In general, egocentric network measures are more loosely representative of sociological constructs than their sociocentric counterparts [12]. For example, egocentric degree does not account for directionality and therefore cannot characterize reciprocity (if A is a friend of B then B is more likely to be a friend of A). Moreover, because the egocentric design used here does not differentiate between different types of triads, it confounds transitivity (A and B are more likely to be friends if C is a friend of both of them) with three-cycles (A is a friend of B who is a friend of C who is a friend of A) and other triadic structures. Nonetheless, egocentric network measures based on survey data are still useful for describing how individual’s networks evolve.

Because closeness is undefined if the ego has no alters (i.e., degree = 0) and transitivity is undefined if they have one or no alters (i.e., degree≤1), we define them to have value 0 if 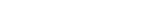 and 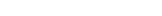 respectively. We include 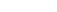 in any model in which closeness is a predictor and 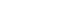 in any model in which transitivity is a predictor. We exclude from the analysis of closeness observations with 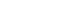 and for the analysis of transitivity we discard observations with 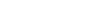


**Table 1:** Regressions of individual outcomes on current and lagged network measures.

Term	BMI		Health		Health behaviors		Pro-social behavior	
	Estimate	95% CI	Estimate	95% CI	Estimate	95% CI	Estimate	95% CI
Network measures								
Degree	0.02	(−0.83, 0.88)	0.08	(−0.11, 0.27)	−0.02	(−0.25, 0.21)	**0.33**	**(0.19, 0.48)**
Closeness	−0.14	(−1.37, 1.10)	0.34	(−0.03, 0.70)	0.38	(−0.09, 0.86)	0.22	(−0.09, 0.54)
Transitivity	−0.04	(−0.86, 0.77)	−0.06	(−0.24, 0.12)	−0.19	(−0.38, 0.01)	−0.09	(−0.23, 0.06)
Lag network measures								
Degree	0.08	(−0.71, 0.88)	0.13	(−0.06, 0.32)	0.00	(−0.22, 0.22)	−0.08	(−0.22, 0.07)
Closeness	0.14	(−1.24, 1.53)	0.08	(−0.32, 0.47)	−0.23	(−0.69, 0.24)	−0.22	(−0.51, 0.07)
Transitivity	0.22	(−0.44, 0.87)	0.07	(−0.12, 0.25)	**0.20**	**(0.01, 0.40)**	0.11	(−0.04, 0.26)
Other predictors								
Wave	0.26	(−0.07, 0.60)	0.02	(−0.05, 0.09)	0.09	(0.01, 0.17)	−0.05	(−0.11, 0.00)
Female	−0.11	(−0.32, 0.10)	0.00	(−0.06, 0.05)	0.04	(−0.03, 0.10)	0.00	(−0.04, 0.04)
Age (10s of years)	0.03	(−0.08, 0.15)	0.01	(−0.01, 0.03)	0.03	(0.01, 0.05)	0.03	(0.02, 0.05)
Lag dependent variable	0.89	(0.81, 0.96)	0.78	(0.76, 0.81)	0.56	(0.53, 0.60)	0.74	(0.72, 0.77)

**Table 2:** Regressions of network measures on lagged health and behavioral traits.

Term	Degree (%)		Closeness (%)		Transitivity (%)	
	Estimate	95% CI	Estimate	95% CI	Estimate	95% CI
Lagged health behaviors						
BMI	**0.15**	**(0.02, 0.27)**	0.02	(−0.05, 0.08)	0.00	(−0.10, 0.10)
Health	0.17	(−0.52, 0.85)	**0.45**	**(0.09, 0.81)**	0.11	(−0.46, 0.68)
Health behavior	0.54	(−0.21, 1.28)	−0.26	(−0.63, 0.11)	0.06	(−0.57, 0.68)
Pro-social behavior	0.38	(−0.52, 1.27)	**0.46**	**(0.02, 0.91)**	0.48	(−0.25, 1.20)
Other predictors						
Wave	−0.88	(−2.59, 0.83)	1.65	(0.81, 2.50)	1.83	(0.20, 3.45)
Female	5.98	(4.38, 7.59)	0.86	(0.08, 1.63)	−2.18	(−3.44, −0.91)
Age (10 s of years)	0.59	(0.02, 1.16)	0.11	(−0.16, 0.37)	0.89	(0.42, 1.36)
Lag dependent variable	0.31	(0.26, 0.35)	0.46	(0.41, 0.51)	0.63	(0.59, 0.67)

Note: When the full model in Equation 5 was fit, (current) pro-social behavior was highly predictive of degree (2.54 percentage-points, 1.39–3.67).

### Statistical Models

We fit a series of models where the ego’s health and behaviors are regressed on the network measures, adjusting for personal characteristics of the ego such as gender and age. In addition, we adjusted for the value of the dependent variable at the previous wave, personal characteristics of the ego (e.g., gender, age), and survey wave. We also fit similar models in which the network measures are regressed on the health and behavioral (HB) measures.

Let 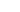 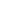 and 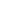 be the vectors of HB (BMI, health, health behavior, prosocial behavior), their network variables (degree, closeness, transitivity), and exogenous control variables (gender, age, survey wave) for individual *i* at survey wave *t* 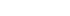 Element *h* of 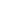 and 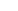 is denoted by 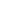 and 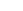 respectively. When applicable, 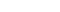 and 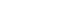 are included in 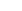 The model for health and behavioral trait *h* regressed on the network measures is
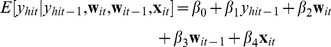
(3)and the model for network measure *k* regressed on the HB is



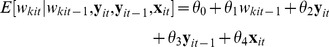
(4)Both (3) and (4) allow the effects of current and lagged predictors to be separated and are jointly useful for detecting directionality of effects. For example, the scenario when 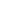 is not significant while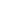 and 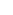 are significant is consistent with network measures having a causal effect on HB but not the converse. In general, if an element of 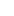 or 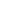 is significant, there is stronger evidence of a causal relationship than if the corresponding element of 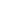 or 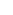 is significant.

**Figure 4:**
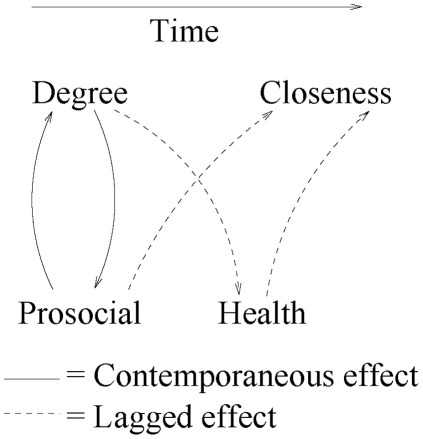
Flow diagram illustrating the primary effects found between network measures and HB. The strongest effect is the contemporaneous bidirectional effect between number of friends (degree) and prosocial behavior (solid line) while the lagged directional effects (dashed lines) were weaker but still statistically significant. Relationships within network measures and within HB are not depicted.

In the event that the effects of current values of the key predictors (network measures or HB) are nearly all non-significant, we consider a reduced model in which they are excluded. In general, lagged models have the advantage over cross-sectional models of being more causally defensible. However, they can lack power.

To investigate the possibility of nonlinearity, we tested whether transitivity-squared was predictive of ego’s outcomes. However, no such effects were found and so we omitted transitivity-squared as a predictor. We also tested whether the turnover in the ego’s peers, defined as
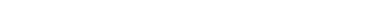
where 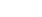 if 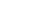 and 0 otherwise, had an impact beyond degree and change of degree. In all instances, estimated effects for 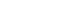 were not significant.

To improve scalability of the estimated regression coefficients, the network measures were scaled to the unit interval when functioning as predictors (as for models of HB) and to percentages when functioning as outcomes. In addition, age was scaled to units of 10 years.

## Results

Of the 6,000 subjects in the sampling frame, a total of 3,232 responded to the first survey. Of these, 2,305 subjects responded to the second survey, and of these 1,809 responded to all three surveys. In response to the name generators (“Who do you spend free time with” and “Who do you discuss important issues with”), we found that Americans identify an average of 4.4±1.8 close social contacts (the average respondent lists 2.2 friends, 0.76 spouses, 0.28 siblings, 0.44 coworkers, and 0.30 neighbors). This corresponds to past work with the GSS [9].

### Change in Network Measures

Between the six-month waves, an ego’s degree increased 31% of the time, stayed the same 27% of the time, and decreased 42% of the time. The transition matrix for ego’s degree (not presented) revealed that unchanged degree occurred most commonly and that there was a clear tendency towards lower degree, especially among egos that began with modest degree (2 to 4 alters).

The distributions of closeness and transitivity measures were very similar between waves with almost equal numbers of increasing and decreasing transitions (Figure 2). Closeness increased 46%, remained the same 8%, and decreased 46% of the time, while transitivity increased 50%, stayed the same 5%, and decreased 45% of the time. All network measures regressed towards the mean (higher values were more likely to decline while lower values were more likely to increase); degree and transitivity were most and least affected, respectively.

### Relationships Among Network Measures

Change in degree and change in closeness have a distinctly negative relationship (Figure 3), and people seem to trade of the number of contacts they have with the closeness of those contacts. Furthermore, increased degree is associated with reduced transitivity, while closeness and transitivity are positively correlated. Thus, as an individual accumulates more alters, the average closeness of their own relationships and of the relationships between the alters in their egocentric network decline. These observations illustrate that correlations often arise in social networks due to natural constraints, even when there is no mathematical constraint. For example, individuals that name more alters may on average have ties of lower average strength due to a limit on the number of very close relationships an individual can maintain. Likewise, across all pairs of alters, average relationship strength is likely to be lower if degree is higher as the relative frequency of pairs with a non-close relationship increases.

### Effects between Egocentric Network and Individual Outcomes

Across the four HB we found that current and lagged network measures were significant predictors in various models (Table 1), suggesting that the model in Equation (4) was appropriate. However, in the models of the three network measures (Equation 5), significant effects were more common for the lagged than the current values of the HB (the strong association of prosocial behavior with degree is the lone exception). Therefore, to simplify interpretation, in these models we excluded the current HB predictor variables and refit them (Table 2).

Table 1 shows that individuals who had more friends (higher degree) were likely to behave more prosocially (estimate: 0.33; CI: (0.19, 0.48)). Because we re-scaled degree to the unit interval to improve the scalability of estimates, the interpretation of the coefficient is that 0.33 more prosocial behaviors are expected for every 8 friends added (approximately 0.04 more behaviors per friend added). Furthermore, when the roles of degree and prosocial behavior were reversed, we also found a strong effect (2.54 percentage-points, 1.39–3.67), the only instance of a network measure having a significant effect under the full model (Equation 5). In both models, the lagged effects are weak, suggesting that the *change* in the predictor is more important than its level. These results imply that the relationship between degree and prosocial behavior is bidirectional, fast-acting, and substantial.

Although the association of lagged transitivity with health behaviors is just significant at the 0.05 level (Table 1), it is of similar magnitude and opposite in sign to that of transitivity. Therefore, the overall effect of transitivity is almost entirely accounted for by change in it, not its level.

In the model of health, the coefficients of the degree terms are positive (0.08 and 0.13) and the confidence interval of lagged degree (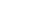 0.32) overlaps 0 by a small amount (Table 1). After excluding degree, lagged degree had a significant association with health (0.18, 0.03–0.34) suggesting that the collinearity of the degree terms reduced the level of lagged degree in the original model. However, the association of lagged health on degree is not significant (Table 2) suggesting that the relationship of degree to health is directional (i.e., not reciprocated) and perhaps varying over time.

We discovered several other directional effects of modest significance (Table 2), including lagged health on closeness (0.45, 0.09–0.81) and lagged prosocial behavior on closeness (0.46, 0.02–0.91). Therefore, a unit increase in health is associated with an expected 0.45 percentage-point increase in average closeness, while adding a prosocial activity leads to a 0.46 percentage-point increase in the closeness of one’s relationships.

The results for the other predictors reveal that age has the strongest association with the outcomes (positive effects on health behaviors and prosocial behavior) and network measures (positive effects on degree and transitivity). As revealed in the exploratory analyses, degree decreased over time while both closeness and transitivity increased. It is interesting to note that females named nearly 6% more friends on average and had stronger relationships, but the relationships among their alters were weaker.

## Discussion

In addition to providing a quantitative description of the changes in individuals’ social networks over time in a national sample, we uncovered interesting relationships among the egocentric network measures, including the negative dependence between degree and either closeness of ties or transitivity among alters. Such dependencies would seem to be a consequence of individuals’ limited capacity to maintain large numbers of close-ties: as our networks become larger, each tie we have to others is expected to weaken. To our knowledge, the phenomena that the greater the number of ties, the less well ones alters know each other, has not been reported previously.

Just as there are certain cognitive limits to the number of individuals one can have as part of one’s social network, [13], [14] it also appears that there are cognitive and temporal considerations for how humans manage their interactions. In particular, we find that the reported average closeness to all friends decreases as the number of one’s friends increases, suggesting an invariant total expenditure on social interaction. An increase of one in the number of close social contacts was associated with a decrease of 0.03 in the average closeness of each individual contact on a scale where 0 =  do not know and 1 =  extremely close. An increase of two close contacts was associated with a decrease in closeness of nearly 0.06 (a substantial reduction on this scale). Because, in prior research, ties are typically modeled as either present or absent, with no strength information, these findings are some of the first of their kind.

In addition, we evaluated a series of regression models relating individuals’ egocentric network measures to their health and behavioral traits. The strongest result we found is the bidirectional association between a respondent’s degree and their prosocial behavior (Figure 4). Moreover, when current network measures were excluded, degree was associated with health, while the reverse is not true (i.e., health was not associated with a subsequent change in the number of friends); this result is consistent with prior work [15]. In addition, being healthier and more prosocial was associated with the development of closer relationships, but the converse was not observed.

Although our principal finding that degree and prosocial behavior are highly predictive of each other is a contemporaneous effect, and thus open to more scrutiny from a causal standpoint than a lagged association of the same significance, we nonetheless raise the possibility that this provides evidence of some effect. Whether degree affects prosocial behavior or the converse, or whether both occur simultaneously, is a question that may only be answerable by a study in which subjects are even more closely followed over time. Across the pairs of individual outcomes and network measures, there were no other bidirectional associations with such a high level of significance.

Considering all results together, the only network measure that consistently predicted health status and also one’s health and prosocial behavior is degree, while both health and prosocial behavior predicted network measures (closeness and degree respectively). These results are consistent with a circular evolution or feedback mechanism among one’s network and one’s health or behavior, possibly reflecting a process that for some or all individuals may tend to an equilibrium position.

Although an egocentric study provides a feasible way of obtaining network information on a large-scale (e.g., by augmenting an existing national survey), it has several limitations compared to a full sociocentric study. First, unlike sociocentric studies, we do not observe the status of the relationship from the perspective of alters. Egocentric data is based purely upon the knowledge, reflection, and recall of the ego, which may be inaccurate – especially when describing the relationship between two alters. Because we do not observe alter’s view of relationships, we are not able to validate the ego self-report, nor are we able to ascertain directionality of relationships [16]. This prevents us from utilizing directionality to distinguish the effect of induction from other phenomena, such as homophilly [17], [18], [19]. However, the analysis is still relevant if we consider the fact that an ego’s *perception* of relationships may be more important than whether or not the perceived relationship is validated via reciprocation by the alter.

Second, without some means of validating the relationship between pairs of alters, relationships elicited via surveys are prone to confound true relationship status with recall – it is likely that relationships with whom an individual has recently been in contact are the most likely to be reported. Therefore, changes in the size or composition of a network may be observed due to changes in the set of alters an individual recently interacted with, even if their true egocentric network is unchanged [20], [21], [22]. If measurement error is random, estimated effects will be attenuated (biased towards 0). Thus, we are more likely to not report a true effect (type II error) than to claim a significant effect when the true effect is 0 (type I error). Such problems with self-reported relationships also apply to sociocentric designs if tie-ascertainment is through self-report. An alternative to recall is to evaluate relationships by monitoring human interactions [23]. Although monitoring might be a more precise way of measuring relationships, or at least to generate a list of potential alters to use in an interview with the ego, in practice such monitoring is likely to involve enormous cost. Clearly, an important area for future work is to study the properties of egocentric networks when relationships are not completely ascertained due to incomplete recollection of ties.

Another limitation is that online surveys have been found to yield less accurate results than in-person interviews, as individuals may answer more mechanically, particularly in completing the response matrix of the relationships between their alters [24]. However, an advantage of performing the survey online is that the expense is much lower and there is no risk of bias from interviewer effects.

Egocentric network studies embedded within population surveys enable population-representative data to be obtained on individuals’ relationships. It is clear that, despite the advantages that sociocentric studies offer, they are not practical on a national scale (except, perhaps, if phone data for a whole nation were used). An understanding of social network structure and its relationship with health and health behaviors can improve understanding of health phenomena such as collateral effects [25], design of healthcare interventions, and evaluation of healthcare policy studies [1]. Egocentric studies, such as that described here, can also provide information about social networks and how they change in ways relevant to health at the national level.
